# Splicing and Polyadenylation of Human Papillomavirus Type 16 mRNAs

**DOI:** 10.3390/ijms18020366

**Published:** 2017-02-09

**Authors:** Chengjun Wu, Naoko Kajitani, Stefan Schwartz

**Affiliations:** Department of Laboratory Medicine, Lund university, 223 62 Lund, Sweden; troy_chengjun.wu@med.lu.se

**Keywords:** human papillomavirus, HPV16, splicing, polyadenylation, hnRNP, SR-protein

## Abstract

The human papillomavirus type 16 (HPV16) life cycle can be divided into an early stage in which the HPV16 genomic DNA is replicated, and a late stage in which the HPV16 structural proteins are synthesized and virions are produced. A strong coupling between the viral life cycle and the differentiation state of the infected cell is highly characteristic of all HPVs. The switch from the HPV16 early gene expression program to the late requires a promoter switch, a polyadenylation signal switch and a shift in alternative splicing. A number of *cis*-acting RNA elements on the HPV16 mRNAs and cellular and viral factors interacting with these elements are involved in the control of HPV16 gene expression. This review summarizes our knowledge of HPV16 *cis*-acting RNA elements and cellular and viral *trans*-acting factors that regulate HPV16 gene expression at the level of splicing and polyadenylation.

## 1. Introduction

Human papillomaviruses (HPV) are ancient viruses that co-evolve with humans and have been infecting humans since the dawn of *Homo sapiens* in the world [1]. They are very well adapted to humans and are successfully infecting a large proportion of the human population, but the vast majority of all HPV infections are asymptomatic [2]. There are more than 200 different human papillomaviruses, all with tropism for epithelial cells [3]. A subset of the HPVs is sexually transmitted and preferentially infect mucosal cells. The latter may be divided into low risk types that are rarely found in cancers, and high-risk types that have been found in primarily anogenital cancers. However, even for the high-risk types, many of which are among the most common HPV types in the human population, is cancer a very rare outcome of the infection. The most common high risk HPV type is HPV16 [4]. It is the most common sexually transmitted HPV-type in the human population, and it is the most common HPV type in anogenital cancers, primarily cancer of the uterine cervix, as well as in tonsillar cancer [4,5]. HPV16 is therefore of particular interest among the HPVs. Posttranscriptional regulation of gene expression in papillomaviruses has been reviewed before [6,7,8], including a summary of cellular proteins that bind HPV mRNAs [9] and alternatively spliced HPV mRNAs [10]. This review will concentrate on the major oncogenic human papillomavirus HPV16, and summarizes our current knowledge of HPV16 gene regulation at the level of RNA processing.

The 50 nm HPV16 virion consists of the viral DNA genome of roughly 8 KB and the capsid proteins L1 and L2 [11]. The viral genome encodes more than six early proteins, produced from multiple alternatively spliced transcripts, and at least two late proteins which include the two capsid proteins L1 and L2 [3]. The main functions of the various HPV16 proteins in the viral life cycle are listed in Table 1. HPVs are strictly epitheliotropic and HPV16 infects primarily mucosal cells. The HPV16 infection starts in mucosal cells in the basal layer of the squamous epithelium and the HPV16 life cycle is intimately linked to the differentiation pathway of the infected cell [2]. Although HPV16 follows the differentiation pathway and depends on both cell proliferation and terminal cell differentiation, it also interferes with cellular functions for its own purpose.

**Figure 1:**
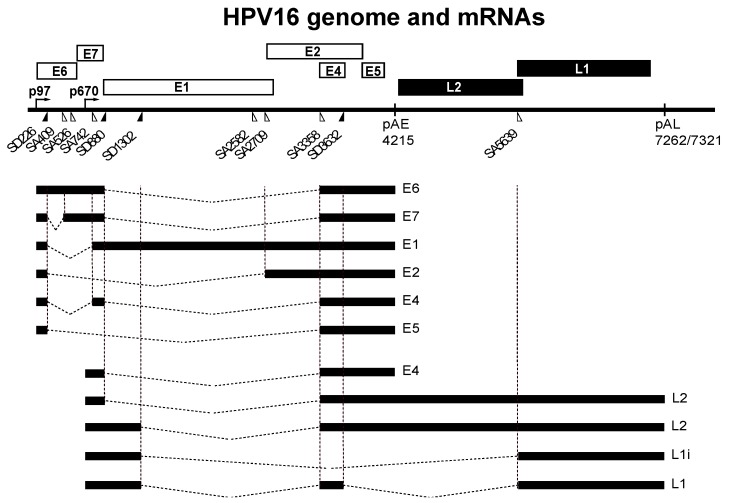
Schematic drawing of the human papillomavirus type 16 (HPV16) genome [3]. Boxes indicate open reading frames, filled triangles represent 5′-splice sites and open triangles represent 3′-splice sites. The early promoter p97 and the late promoter p670 are indicated, early and late polyadenylation signals pAE and pAL are indicated. A selection of alternatively spliced HPV16 mRNAs are shown below the HPV16 genome. L1 and L1i represent two alternatively spliced L1 mRNAs.

The HPV16 life cycle can be divided into an early stage in which the HPV16 genomic DNA is replicated, and a late stage in which the HPV16 structural proteins are synthesized and virions are produced [12,13] (Table 1). A strong coupling between the viral life cycle and the differentiation state of the infected cell is highly characteristic of all HPVs [2]. Initially E6 and E7 proteins drive cell proliferation by binding to and degrading p53 and pRb [14,15]. Additional targets of E6 and E7 have been shown to be important as well [14,15]. The role of E6 and E7 is to induce an intracellular environment that is optimal for replication of the HPV16 DNA genome. As HPV16 lacks a DNA polymerase, the HPV16 E1 and E2 proteins are key factors during replication of HPV16 DNA and bring together HPV16 DNA genome and the cellular DNA polymerase to initiate DNA synthesis [16,17,18]. The HPV16 E2 protein is a multifunctional protein that binds directly to HPV16 DNA as well as cellular chromatin and participates in viral DNA replication, partitioning, transcription and polyadenylation [17,19]. As the HPV16 genome replicates to high levels, E2 accumulates in the HPV16 infected cells. E2 bind to the HPV16 early promoter and inhibits transcription from the HPV16 early promoter p97 (Figure 1), thereby shutting down expression of the promitotic and antiapoptotic E6 and E7 proteins. This crucial step allows the HPV16 infected cells to re-enter the cellular differentiation program and to differentiate terminally and reach the very top of the infected mucosal epithelium. Terminal cell differentiation is essential for activation of the HPV16 late promoter p670 from which all HPV16 proteins but E6 and E7 can be produced. The late promoter drives expression of high levels of E4 protein, which precedes expression of the HPV16 late structural L1 and L2 proteins [13]. Terminal cell differentiation is also accompanied by altered splicing and polyadenylation functions in the HPV16 infected cells, which results in inhibition of the HPV16 early polyA signal and read-through into the late region to produce L2 mRNAs, and activation of HPV16 late spliced sites SD3632 and SA5639 which generates L1 mRNAs. Terminal cell differentiation is required for induction of HPV16 late gene expression, L1 and L2 capsid protein production and for production of virus particles that are shed from the top of the epithelium.

**Figure 2:**
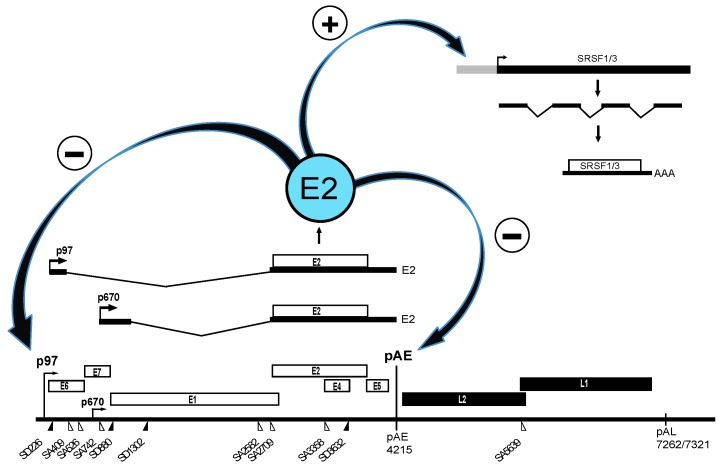
The HPV16 E2 proteins can be produced from two mRNAs generated either from the HPV16 early promoter p97, or the late promoter p670 [3]. These mRNAs are indicated above the schematic drawing of the HPV16 genome. The E2 protein affects HPV16 gene expression by inhibiting transcription from the HPV16 early promoter p97, which inhibits E6 and E7 expression [17], and by inhibiting the HPV16 early polyadenylation signal pAE, which promotes HPV16 late gene expression by inducing read-through into the HPV16 late region [22]. E2 could potentially also indirectly affect HPV16 RNA processing by activating expression of genes encoding serine/arginine rich (SR) proteins [23,24]. A direct induction of HPV16 late gene expression by E2 occurs when HPV16 affects the conformation of the polyadenylation complex that forms on pAE, thereby inhibiting pAE [22]. E2 therefore could function as a switch from HPV16 early gene expression to HPV16 late gene expression.

At the level of gene expression, the HPV16 early stage is characterized by transcription from the HPV16 early promoter termed p97 (Figure 1) [3,20]. This promoter generates a number of alternatively spliced mRNAs that are all polyadenylated at the early polyadenylation signal pAE (Figure 1) [3,10]. These mRNAs have the potential produce the majority of all HPV16 early proteins (Figure 1). While the E6 and E7 proteins drive cell proliferation at the early stage, terminal differentiation is finally required for induction of L1 and L2 gene expression, production of L1 and L2 proteins and viral particles. When high levels of E2 protein accumulate in the HPV16 infected cells, the E2 protein binds to the p97 promoter to shut it down [19,21], turning off E6 and E7 expression and allowing the cell to re-enter the differentiation program (Figure 2). As a result of cell differentiation, transcription from the late HPV16 promoter p670 is activated [20]. At the same time, the HPV16 E2 protein inhibits the HPV16 early polyadenylation signal to allow for read-through into the late region of the HPV16 genome, paving the way for production of L1 and L2 mRNAs encoding the HPV16 capsid proteins (Figure 2) [22]. These mRNAs are polyadenylated at the late polyA signal pAL and use late splice sites that are specific for the HPV16 late mRNAs [3,10]. High levels of E2 induces a switch from the early to the late HPV16 gene expression program, that eventually results in the production of HPV16 virions at the very top of the epithelium. In summary, the switch from the HPV16 early gene expression program to the late requires a promoter switch, a polyA signal switch and a shift in alternative splicing. A number of cellular factors is involved in these processes. To understand the HPV16 life cycle it is of major importance to understand how it switches from the early to the late stage and how this switch is regulated at the level of splicing and polyadenylation.

Although HPV16 uses the cellular transcription-, splicing- and polyadenylation-machineries to express viral genes, there are a number of differences between HPV16 genes and cellular genes. The nucleotide composition of the HPV16 genome is skewed towards A and T, and consequently mRNAs are more AU-rich than the average cellular mRNAs and may attract different sets of RNA binding proteins and/or may be differently processed. Secondly, the majority of the transcribed region of the HPV16 genome is protein coding, often with overlapping coding regions (Figure 1). The early and late untranslated regions (UTRs) constitute rare exceptions by not coding for protein. Consequently, RNA sequences that are spliced out on some mRNAs and would constitute introns or non-coding, would constitute exons on other HPV16 mRNAs. Finally, the length of HPV16 exons extends beyond the average 100 nucleotides in length observed for exons in cellular genes and ranges from approximately 120 nucleotides to almost 4 KB. Therefore, the larger exons would include multiple unutilized splice sites, regulatory RNA elements and both exonic and intronic sequences. These properties of HPV16 add an additional level of complexity to the control of HPV16 gene expression at the level of RNA processing.

## 2. HPV16 Gene Regulation at the Levels of RNA Splicing and Polyadenylation

### 2.1. Upstream Region in HPV16

The upstream region of the HPV16 genome contains two 5′-splice sites named SD226 and SD880, and three 3′-splice sites named SA409, SA526 and SA742 (Figure 1) [3,10]. With the exception of SD880, mutations in these splice sites appear to affect usage only of the neighbouring sites in this region suggesting that splice sites SD226 and SA409, SA526 and SA742 are highly connected and compete with each other rather than with downstream HPV16 splice-sites [25]. Splicing appears to be regulated by heterogenous nuclear ribonuclear protein A1 (hnRNP A1) and Sam68, but binding sites for these RNA binding proteins have not yet been identified [26]. hnRNP A1 is an RNA binding protein that belongs to the hnRNP-family of proteins and it often binds splicing silencer elements on cellular mRNAs to inhibit splicing [27]. Sam68 belongs to the STAR family of proteins that link signalling pathways to RNA processing [28]. It would be of interest to determine the target sites of these RNA binding proteins on the HPV16 early mRNAs since these factors control expression of the two HPV16 oncogenes E6 and E7 [29] and as such could affect the pathogenic properties of HPV16. Indeed, variant genomes of HPV16 may display different splicing efficiencies in this region [30,31] and hnRNP A1 is overexpressed in high-grade cervical lesions and cervical cancer cells that contain HPV16 DNA [32].

### 2.2. Middle Region in HPV16

**Figure 3:**
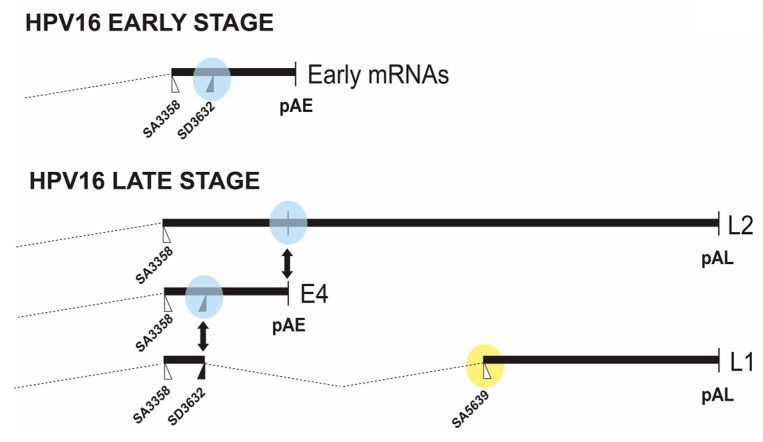
Schematic drawing of the middle and late regions of the HPV16 genome. **Upper** panel: splicing to HPV16 3′-splice site SA3358 followed by polyadenylation at the HPV early polyadenylation signal pAE at the early stage of the HPV16 infection. The suppressed HPV16 late 5′-splice site SD3632 is encircled [6]; **Lower** panel: at the late stage of the HPV16 infection the three late mRNAs L2, L1 and E4 co-exist. This is the result of partial inhibition of pAE, which causes read-through into the late region and polyadenylation at pAL to produce the L2 mRNAs. Activation of the two exclusively late splice sites SD3632 and SA5639 results in production of L1 mRNAs, while a fraction of the mRNAs that are polyadenylated at pAE produces E4 mRNAs. The E4 protein is produced at both early and late stages of the HPV16 life cycle. The regulated HPV16 late 5′-splice site SD3632 and the early polyA signal pAE are encircled in blue and HPV16 late 3′-splice site SA5639 in yellow.

The middle region of the HPV16 genome contains splice sites and a polyadenylation signal (pAE) that are subject to regulation. At an early stage of the HPV16 life cycle, the HPV16 3′-splice site SA3358 connects with the early polyadenylation signal pAE to produce all early mRNAs except E1 and E2 mRNAs, thus producing E6, E7, E4 and E5 mRNAs (Figure 1). As the HPV16-infected cell differentiates and the early promoter p97 is shut down and the late promoter p670 is induced, the usage of SA3358 appears to increase, and is now utilized for production of E4, L2 and L1 mRNAs (Figure 3). HPV16 3′-splice site SA3358 is therefore used during both early and late stages of the HPV16 viral life cycle. The HPV16 E4 protein is therefore part of both the early and the late HPV16 gene expression programs. During the early stage, SA3358 efficiently connects with the early polyadenylation signal pAE to produce mRNAs that end at pAE (Figure 3). This efficient connection prevents read-through into the late region and inhibits utilisation of the exclusively late HPV16 5′-splice site SD3632 that is located in between SA3358 and pAE (Figure 3). Measures to prevent HPV16 late gene expression at an early stage of the HPV16 life cycle include active suppression of SD3632 as well as positive stimulation of pAE by multiple elements located upstream and downstream of pAE. The HPV16 3′-splice site SA3358 is therefore a key player in the switch from HPV16 early to late switch by its association with either the early polyadenylation signal at the early stage of the infection, and late splice site SD3632 and both pAE and pAL at the late stage of the infection (Figure 3).

#### 2.2.1. Regulation of HPV16 SA3358

The HPV16 3′-splice site SA3358 has an upstream polypyrimidine tract that is highly interrupted by purines and as such is predicted to be inefficient [3]. However, HPV16 SA3358 is one of the most frequently used splice sites on the HPV16 genome [33]. It is used during the entire life cycle and it is used for production of both early (e.g., E6, E7) and late (e.g., L1 and L2) mRNAs (Figure 1 and Figure 3). The poor homology to a consensus splice site therefore suggests that HPV16 SA3358 is subject to regulation rather than being inefficiently used, since a weak splice site can be controlled in contrast to constitutively active splice sites.

**Figure 4:**
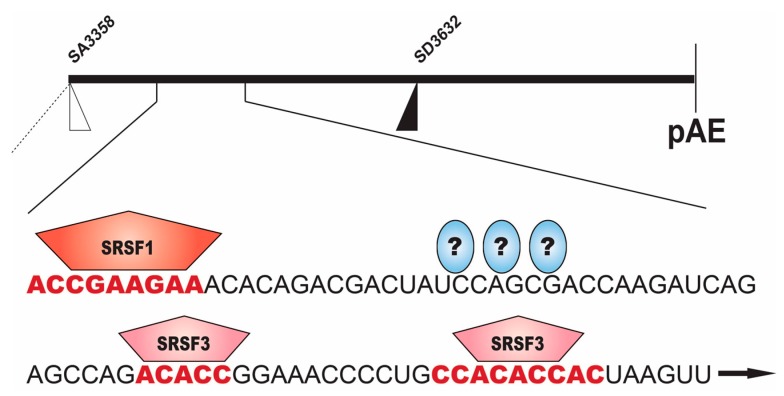
Schematic drawing of the middle region of the HPV16 genome ranging from the HPV16 3′-splice site SA3358 to the early polyadenylation signal pAE. The suppressed HPV16 late 5′-splice site SD3632 is indicated [41]. The blow up shows the splicing enhancer that is required for splicing to SA3358 [34,35,36]. Binding sites for the splicing factors serine/argine rich splicing factors 1 and 3 (SRSF1 and SRSF3) are indicated in red [6,7,9,35,36,37]. Question marks indicate unidentified cellular proteins that bind to the splicing enhancer.

The HPV16 3′-splice site is controlled by strong splicing enhancers located downstream of the splice site itself (Figure 4) [34]. Deletions or point mutations introduced in this enhancer elements, reduce splicing to SA3358 and redirect splicing to downstream, competing splice sites [34,35,36,37]. The splicing enhancer is a bipartite element consisting of an 8-nucleotide purine-rich binding site for serine/argine rich splicing factor 1 (SRSF1), and a downstream AC-rich region to which SRSF3 binds and binding sites have been mapped (Figure 4) [35,36,37]. SR-proteins belong to the SR-protein family of splicing factors [38]. In addition, SRSF9 has been shown to bind directly to splicing enhancer sequences downstream of SA3358, but the exact binding sites were not mapped [39]. Overexpression or increased levels of either SRSF3 or SRF9 appears to reduce splicing to SA3358 [23,37,39]. In HPV18, knock down of SRSF3 reduces splicing to the 3′-splice site that corresponds to HPV16 SA3358, while E2 and L1 mRNAs increase [40]. There may be genuine differences in the regulation of HPV16 and HPV18 gene expression. Since SR-proteins are regulated by phosphorylation and the unphosphorylated and phosphorylated forms may compete with each other, it remains to be determined how exactly SR-proteins control HPV16 SA3358. It is reasonable to speculate that a splice site such as HPV16 SA3358 that is connected to both polyadenylation signals (pAE and pAL) and 5′-splice site SD3632 depending on the stage of the HPV16 life cycle (Figure 3), will be regulated by multiple factors and that not all these factors have been identified yet.

#### 2.2.2. Regulation of HPV16 SD3632

**Figure 5:**
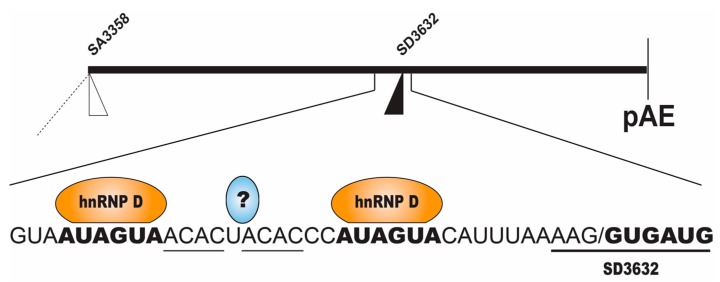
Schematic drawing of the middle region of the HPV16 genome with the HPV16 3′-splice site SA3358 used by both early and late mRNAs, the exclusively late 5′-splice site SD3632 and the early polyadenylation signal pAE. The blow up shows the suppressed HPV16 late 5′-splice site SD3632 with upstream splicing silencer elements [34] consisting of two AUAGUA-motifs that bind heterogenous nuclear ribonuclear protein D (hnRNP D) and two ACAC-motifs that interact with a yet unknown cellular factor [6,9,41]. Question mark indicates unidentified cellular proteins that bind to the splicing enhancer. Binding sited for hnRNP D and the intronic region of HPV16 late 5′-splice site SD3632 are in boldface, and entire SD3632 is underlined.

The HPV16 late 5′-splice site SD3632 is suppressed at the early stage of the HPV16 life cycle (Figure 3), including mitotic cells, high grade cervical lesions and cancer cells. This splice site is strongly suppressed by a sequence located immediately upstream of 5′-splice site SD3632 (Figure 5) [34,41]. The splicing inhibitory sequence encodes two AUAGUA-motifs and two ACAC-sequences (Figure 5) [41]. The two AUAGUA motifs binds specifically to hnRNP D, whereas the factor binding to the AC-rich motifs is yet unidentified (Figure 5) [41]. hnRNP D belongs to the hnRNP family and regulates splicing of cellular and viral mRNAs in addition to its ability to bind AU-rich RNA instability elements [27]. The specific binding of hnRNP D to splicing silencer sequences correlates with inhibition of splice site SD3632 [41]. The complex of proteins binding to the splicing silencer element also contains hnRNP A2B1. Therefore, hnRNP D proteins are suppressors of HPV16 SD3632.

#### 2.2.3. Regulation of HPV16 Early PolyA Signal pAE

**Figure 6:**
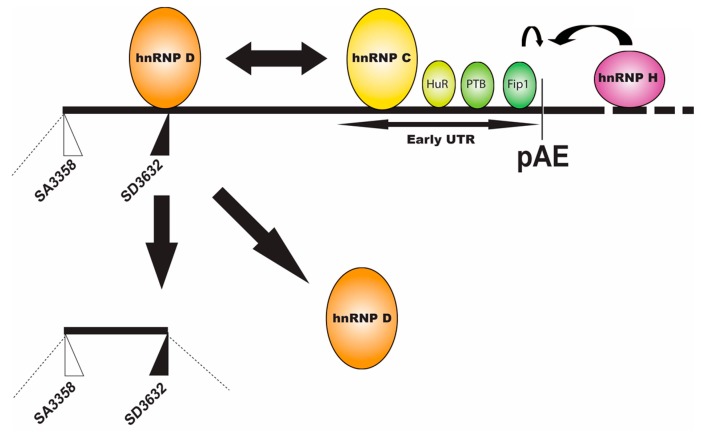
Schematic drawing of the middle region of the HPV16 genome. HPV16 3′-splice site SA3358, used by both early and late mRNAs is indicated. The exclusively late 5′-splice site SD3632 with hnRNP D binding to upstream splicing silencer elements to suppress SD3632 [41]. heterogenous nuclear ribonuclear protein C (hnRNP C), HuR, polypyrimidine tract binding protein (PTB) and Fip1 binding to the HPV16 early untranslated region (UTR) are indicated [6,9,22,43,48,51]. hnRNP H binds to sequences downstream of the HPV16 early polyadenylation signal pAE to promote polyadenylation at pAE [6,9,46,47]. Double arrow represents the interactions between hnRNP C that binds to the HPV16 early UTR, and hnRNP D that binds to the splicing silencers upstream of SD3632 [9,48]. The interaction of hnRNP C with hnRNP D activates HPV16 late 5′-splice site SD3632 and induces splicing from SD3632 to SA5639 and thereby activates production of the spliced, major late L1 mRNAs [9,48].

Regulation of the HPV16 early polyA signal pAE is key to the entry into the late stage of the HPV16 life cycle since inhibition of pAE allows read-through into the L2 and L1 coding regions and polyadenylation at the late polyA signal pAL (Figure 3). Mutational inactivation of pAE results in polyadenylation at nearby, upstream A-rich regions, demonstrating that adjacent regulatory elements promote polyadenylation in this region of the HPV16 genome [42,43]. Interestingly, the upstream HPV16 3′-splice site SA3358 appears to have a major impact on the HPV16 early polyA signal. If HPV16 SA3358 is not used, mutated or deleted, polydenylation does not occur at pAE [34]. This is of particular interest in the light of the fact that SA3358 is used throughout the HPV16 replication cycle, which opens the possibility for regulation of pAE through control of SA3358. That is, HPV16 SA3358 may be controlled by different factors during the HPV16 life cycle, and these factors may control different RNA processing events. The HPV16 early polyA signal pAE is controlled by upstream and downstream sequences (Figure 6) [43,44,45,46,47]. The upstream UTR is AU-rich and has a positive effect on pAE, but may also exert a more general regulatory function during the HPV16 life cycle [43,48]. It interacts with a number of cellular factors including polypyrimidine tract binding protein (PTB), RALYL, heterogenous nuclear ribonuclear protein C (hnRNP C), HuR and Fip1 (Figure 6) (unpublished data and [34,48]). PTB, RALYL and hnRNP C belong to the hnRNP family of proteins and all bind to pyrimidine rich or U-rich sequences to regulate polyadenylation and splicing [27] whereas HuR binds AU-rich RNA instability elements [49] and Fip1 is part of the polaydenylation complex [50]. Overexpression of PTB causes read-through at pAE, suggesting a regulatory function for PTB in HPV16 early polyadenylation [51]. Members of the hnRNP-family of hnRNPs (hnRNP C1 and RALYL) are also believed to control HPV16 pAE, but appear to play a more complex role as they also activate SD3632 in an HPV16 early UTR-dependent manner (Figure 6) [48]. Since HPV16 early polyadenylation and utilization of SD3632 are two mutually exclusive events, hnRNP C1 may be the switch between pAE and SD3632, a role that is likely to involve the hnRNP D protein that binds at SD3632 and suppresses this splice site [48].

RNA sequences downstream of HPV16 pAE also control the activity of the pAE [46,47]. This function is conserved between HPV16 and HPV31 [44,45]. In HPV16, these elements encode multiple triple-G motifs and interact with hnRNP H as well as CstF-64 [46,47], and in HPV31 binding to CstF-64 has been reported (Figure 6) [44,45]. hnRNP H belongs to the hnRNP family of proteins and binds G-rich sequences to control RNA splicing and polyadenylation [27] whereas CstF-64 belongs to the core polyadenylation complex [50]. Since levels of both CstF-64 and hnRNP H are affected by cell differentiation, these factors may modulate HPV16 pAE to control access to the late region of the HPV16 genome [44,45,46,47].

Perhaps most importantly, the HPV16 E2 protein itself is involved in the control of pAE (Figure 2) [22]. The HPV16 E2 protein is generally believed to induce entry into the late stage of the HPV16 life cycle by shutting down transcription of the HPV16 early genes [17,19,52,53,54]. When sufficient quantities of E2 have accumulated in the HPV16-infected cells, the HPV16 early promoter p97 is supressed and E6 and E7 expression is downregulated, which paves the way for cellular differentiation and induction of HPV16 late gene expression (Figure 2). HPV5 E2 has an serine/arginine rich (RS)-domain that interacts with SR-proteins [55]. HPV16 E2 lacks an RS-domain but still interacts with SR-proteins [56] and activates expression of cellular SR-proteins [23,24], which may affect SA3358 through the downstream enhancer to increase E4, L1 and L2 expression. HPV16 E2 also directly inhibits the HPV16 early polyadenylation signal pAE to allow for read-through into the late region of the HPV16 genome (Figure 2) [22]. High levels of HPV16 induces HPV16 late gene expression by modulating the conformation of the polyadenylation complex that forms at HPV16 pAE, an effect mediated by the activation domain of the E2 protein [22]. Since the E2 protein also binds RNA [56], it is not unlikely that E2 affects RNA processing of cellular mRNAs as well [57].

### 2.3. Downstream Region in HPV16

#### 2.3.1. Regulation of HPV16 SA5639

**Figure 7:**
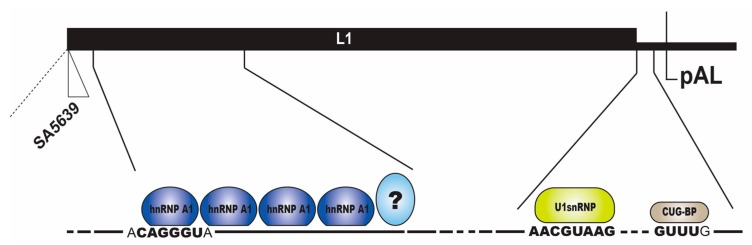
Schematic drawing of the late region of the HPV16 genome with the HPV16 3′-splice site SA5639 and the late polyadenylation signal pAL and the late untranslated region (UTR). The blow up shows the splicing silencer elements downstream of the suppressed HPV16 late 3′-splice site SA5639 [6,59,60]. These RNA elements consist of purine-rich sequences that bind hnRNP A1 [6,9,58,59,60]. Question marks indicate unidentified cellular proteins that bind to the splicing silencer. In addition to the splicing silencer elements downstream of SA5639, the HPV16 late mRNAs encode a negative regulatory element in the HPV16 late untranslated region [62]. The late UTR of many HPVs has an inhibitory function [63]. These elements consist of 5′-splice site-like sequences that bind to U1 small nuclear ribonucleoprotein (snRNP) to inhibit HPV16 late polyadenylation [64,65], and GU-rich elements that bind CUG-triplet repeat RNA binding protein (CUG-BP) [66], and other proteins [62]. Binding sites for hnRNP A1, U1 snRNP and CUG-BP are in boldface.

HPV16 late 3′-splice site SA5639 is located immediately upstream of the L1 ATG and is the most important splice site for generation of mRNAs encoding the major HPV16 capsid protein L1 (Figure 1 and Figure 3) [3,10]. The HPV16 splice site SA5639 conforms well to a consensus 3′-splice site with a relatively long, uninterrupted polypyrimidine tract which predicts high functionality. However, HPV16 is strongly suppressed in mitotic cells and cancer cells (Figure 3). The L1 coding sequences located downstream of HPV16 SA5639 encode multiple splicing silencer elements that are associated with the AU-richness of the HPV16 coding sequences (Figure 7) [58,59,60]. Indeed, reducing the AU-richness of the HPV16 L1 coding region activates the suppressed SA5639 splice site [59,60,61]. These splicing silencer elements bind cellular protein hnRNP A1 and hnRNP A2/B1 (Figure 7)**,** interactions that are lost when the AU-richness is reduced by mutagenesis and splice site SA5639 is activated, suggesting that hnRNP A1 and A2/B1 are major suppressors of HPV16 late 3′-splice site SA5639 (Figure 7) [58,59,60]. However, other yet unidentified factors contribute as well (Figure 7).

#### 2.3.2. Negative Regulatory Elements in the HPV16 Late Untranslated Region

The vast majority of all HPVs appear to be endowed with a negative regulatory element in the late untranslated region [62,63]. Indeed, this property extends to bovine papillomavirus type 1 (BPV-1), suggesting strong conservation and important function [65,67,68]. The HPV16 late UTR is GU-rich and encodes sequences homologous to 5′-splice sites that interact with U1 snRNP [64,65,69], a property shared with BPV-1 [65], and a U-rich region with GUUUG-motifs that resemble RNA instability motifs AUUUA (Figure 7). The U-rich region has been reported to interact with multiple cellular factors with diverse functions in splicing and polyadenylation including SRSF1, a member of the SR-proteins family that often has a positive effect on splicing [38], hnRNP A1 that belongs to the hnRNP family of proteins that often have a negative effect on splicing [27], HuR that binds AU-rich RNA instability elements on cellular mRNAs [49], U2AF65 that binds to and defines 3′-splice sites on cellular mRNAs [38], CstF64 that belongs to the cellular polyadenylation complex [50] and CUG-BP [63,66,70,71,72,73], while the splice site-like sequences interact with U1 snRNP1 and inhibit polyadenylation through interactions between the U1–70K component of U1 snRNP1 and polyA-polymerase (Figure 7) [64,65]. An AU-rich RNA instability element and translation inhibitor in the late UTR of the cutaneous HPV1 binds specifically and directly to HuR, hnRNP C1 and polyA binding protein [74,75,76]. HuR and hnRNP C binds to AU-rich RNA instability elements on cellular mRNAs [27,49] but binding of HuR to the HPV16 late UTR has been controversial [66]. CstF-64, U2AF65 and HuR appear to interact with late UTR sequences on HPV31 as well [69]. In addition to suppressing HPV16 late gene expression at the early stage of the viral life cycle, the late UTR element may also serve as a landing pad for cellular factors that mediates nuclear export of the late viral mRNAs [73]. Indeed, the human immunodeficiency virus type 1 (HIV-1) Rev protein, that is required for nuclear export of unspliced and partially spliced HIV-1 mRNAs [77], can overcome the inhibitory effect of the BPV-1 late UTR [68], the HPV1 late UTR [78] and the HPV16 late UTR [79]. However, the exact role of the late UTR element in the HPV16 life cycle remains to be determined.

## 3. Future Perspectives—Control of HPV16 RNA Processing through Transcription Coupled Transcription and Epigenetics

Chromatin associated factors are in an excellent position to control splicing and polyadenylation of HPV16 mRNAs as they are synthesized by RNA polymerase II. Since the HPV16 E2 protein is chromatin associated and possesses DNA binding activity with specific binding sites in the HPV promoter region, one may speculate that E2 binding to the HPV promoter affects the conformation of the transcription complex, in particular its association with various RNA processing factors [17,19,53]. HPV16 E2 is also an RNA binding protein and is in an excellent position to connect transcription and RNA processing [22,56]. The HPV16 genome displays differences in DNA methylation pattern [80] suggesting a role of DNA methylation in the control of HPV16 gene expression. However, we did not observe differences in HPV16 RNA processing in response to DNA methyl transferase inhibitors [81]. In contrast, inhibitors of histone de-acetylases affected HPV16 RNA processing indicating that histone modifications on the HPV16 genome may contribute to control of HPV16 gene expression [81]. Histone modifications are unevenly distributed over the HPV16 and HPV18 genomes and may contribute to gene regulation [82]. Furthermore, a connection between binding of the DNA-binding protein and essential chromatin regulator CCCTC-binding factor (CTCF) to HPV18 DNA and the splicing of HPV18 mRNAs strongly support the idea of interactions between HPV DNA or chromatin-associated factors and factors that process the HPV mRNAs [83,84]. This would be a particularly cost-effective way of controlling gene expression in a small DNA tumour virus such as HPV and is likely to play an important role in the control of HPV16 gene expression.

## Figures and Tables

**Table 1:** Function of HPV16 proteins.

HPV16 Protein	Protein Function
E1	Replication of viral DNA
E2	Replication of viral DNA
E4	Assists at egress of virus
E5	Promitotic function
E6	Antiapoptotic function
E7	Promitotic function
L1	Major capsid protein
L2	Minor capsid protein
